# Effects of glyphosate residues and different concentrate feed proportions in dairy cow rations on hepatic gene expression, liver histology and biochemical blood parameters

**DOI:** 10.1371/journal.pone.0246679

**Published:** 2021-02-12

**Authors:** Ann-Katrin Heymann, Karina Schnabel, Fabian Billenkamp, Susanne Bühler, Jana Frahm, Susanne Kersten, Liane Hüther, Ulrich Meyer, Dirk von Soosten, Nares Trakooljul, Jens Peter Teifke, Sven Dänicke

**Affiliations:** 1 Institute of Animal Nutrition, Friedrich-Loeffler-Institut (FLI), Federal Research Institute for Animal Health, Braunschweig, Germany; 2 Institute of Genome Biology, Leibniz Institute for Farm Animal Biology (FBN), Dummerstorf, Germany; 3 Department of Experimental Animal Facilities and Biorisk Management, Friedrich-Loeffler-Institut (FLI), Federal Research Institute for Animal Health, Greifswald-Insel Riems, Germany; West Virginia University, UNITED STATES

## Abstract

Glyphosate (GLY) is worldwide one of the most used active substances in non-selective herbicides. Although livestock might be orally exposed via GLY-contaminated feedstuffs, not much is known about possible hepatotoxic effects of GLY. As hepatic xenobiotic and nutrient metabolism are interlinked, toxic effects of GLY residues might be influenced by hepatic nutrient supply. Therefore, a feeding trial with lactating dairy cows was conducted to investigate effects of GLY-contaminated feedstuffs and different concentrate feed proportions (CFP) in the diets as tool for varying nutrient supply to the liver. For this, 61 German Holstein cows (207 ± 49 days in milk; mean ± standard deviation) were either fed a GLY-contaminated total mixed ration (TMR, GLY groups, mean GLY intake 122.7 μg/kg body weight/day) or control TMR (CON groups, mean GLY intake 1.2 μg/kg body weight/day) for 16 weeks. Additionally, both groups were further split into subgroups fed a lower (LC, 30% on dry matter basis) or higher (HC, 60% on dry matter basis) CFP resulting in groups CON_HC_ (n = 16), CON_LC_ (n = 16), GLY_HC_ (n = 15), GLY_LC_ (n = 14). Blood parameters aspartate aminotransferase, γ-glutamyltransferase, glutamate dehydrogenase, cholesterol, triglyceride, total protein, calcium, phosphorus, acetic acid and urea and histopathological evaluation were not influenced by GLY, whereas all mentioned parameters were at least affected by time, CFP or an interactive manner between time and CFP. Total bilirubin blood concentration was significantly influenced by an interaction between GLY and CFP with temporarily elevated concentrations in GLY_HC_, whereas the biological relevance remained unclear. Gene expression analysis indicated 167 CFP-responsive genes, while seven genes showed altered expression in GLY groups compared to CON groups. Since expression changes of GLY-responsive genes were low and liver-related blood parameters changed either not at all or only slightly, the tested GLY formulation was considered to have no toxic effects on the liver of dairy cows.

## Introduction

Glyphosate (GLY; N-phosphonomethylglycine) is one of the most used active substances in herbicides worldwide [1]. Since its introduction as a non-selective herbicide in 1974, possible side-effects of GLY concerning human and animal health have been controversially discussed in the literature [1, 2]. Due to the intensive use in agriculture worldwide, GLY residues can be detected in the environment [3], food [4] and animal feed such as dairy cow rations [5]. The daily GLY exposure of dairy cows was shown to vary between 0.08 and 6.7 mg GLY [5]. According to von Soosten et al. [5], 8 ± 3% of daily consumed GLY is excreted via urine, while 61 ± 11% of consumed GLY is found in feces. Consequently, most GLY passes the digestive tract unmetabolized. Differences between GLY intake and excretion might be result from ruminal degradation [5]. Although ruminal absorption capacity and systemic absorption of GLY appear to be low [5], GLY residues were detected in different organs such as liver, intestine or muscles of German dairy cows [6]. In this context, the liver is of special interest, since next to its key role in energy metabolism, it is responsible for the degradation and excretion of xenobiotics like herbicides [7, 8]. Mesnage et al. [9] detected changes in hepatic gene expression for more than 4000 genes in rats after oral GLY-treatment. According to the authors, these results correlate with observations of hepatic histopathological changes such as necrotic foci [10] and nucleolar disruption of hepatocytes [9] upon dietary GLY-exposure in rats. In addition, other authors reported increased activities of aspartate aminotransferase (AST) and γ-glutamyltransferase (GGT) in the blood of dietary GLY-treated rats [11] and mice [12], which could be indicative for hepatic alterations or damages [11, 12]. Hepatotoxic effects of GLY were examined *in vitro* in human liver cells [13] or *in vivo* in mice [12], rats [11] and fish [14, 15]. However, there is a lack of real-life scenarios and consequently little is known about hepatotoxic effects of GLY on livestock. To address this lack of information and in order to avoid artificial GLY-exposure conditions, this study was designed with regard to a worst-case exposure scenario according to legal applications in Europe [16]. Furthermore, different concentrate feed proportions (CFP) were used to investigate whether putative GLY effects are depending on energy and nutrient supply to the liver since xenobiotic metabolism might be affected by nutrients [17]. In order to address these questions and investigate the interactions between GLY and CFP, a feeding trial was conducted comprising four feeding groups (14–16 animals/group) arranged in complete two by two factorial design. First results of this study showed no adverse effects of the tested GLY formulation on performance, energy metabolism, health characteristics and hematological parameters [18, 19]. In order to get further insights, putative hepatotoxicity of GLY at different hepatic nutrient status was addressed in the present investigations.

## Materials and methods

The experiment was conducted in accordance with the German Animal Welfare Act at the experimental station of the Institute of Animal Nutrition, Friedrich-Loeffler-Institut (FLI), in Braunschweig, Germany and was approved by the Lower Saxony State Office for Consumer Protection and Food Safety (LAVES, 33.19-42502-04-14/1736).

### Animal trial

A detailed description of the experiments and procedures for this study was published together with results concerning the effects of GLY residues and different CFP on performance, energy metabolism, health characteristics as well as hematological parameters and oxidative stress [18, 19]. In short, 61 lactating German Holstein cows (207 ± 49 d in milk; parity of 2.8 ± 1.9 (S1 Table), mean ± standard deviation) received a total mixed ration (TMR) consisting of 30% maize silage, 30% grass silage, 40% concentrate on dry matter (DM) basis for one week (week 0) as an adaption period. After this period cows were assigned to groups according to their number of lactations, mean of body weight, feed intake and fat corrected milk as described [19]. The groups were fed ad libitum with a GLY-contaminated TMR (GLY groups) or with a control TMR (CON groups) for 16 weeks. GLY and CON groups were further split into subgroups fed with different CFP. Low CFP groups (LC) received a TMR consisting of 21% maize silage, 42% grass silage, 7% straw and 30% concentrate (LC) on DM basis, while high CFP groups (HC) were fed with a TMR composed of 11% maize silage, 22% grass silage, 7% straw and 60% concentrate based on DM (HC) [19]. This resulted in four groups CON_HC_ (n = 16), CON_LC_ (n = 16), GLY_HC_ (n = 15) and GLY_LC_ (n = 14). The feed was produced at the experimental station of the FLI. The GLY-contaminated diets contained peas, wheat grain and straw contaminated by residues of the GLY formulation Roundup Record^®^ (007525-60/MOT), Monsanto, Agrar Deutschland GmbH (Düsseldorf, Germany) based on a pre-harvest treatment of plants according to regulation (EC) No. 396/2005. This formulation with GLY as the active ingredient (720 g GLY/kg GLY solution) in combination with a surfacting agent, another not specified co-formulant and sodium sulfite was used as water-soluble granulate. In GLY groups, straw was the main GLY source [19]. During the trial, individual daily feed intake was measured by weighing troughs (Insentec, B.V., Marknesse, The Netherlands). Ingredients and chemical composition of the feed is described in detail by Schnabel et al. [19].

### Sample collection

As described [19], samples of straw and concentrates were taken once a week and pooled to a collective sample every four weeks, while maize and grass silage were collected twice a week and pooled to a collective sample every four weeks.

Liver tissue samples of 32 animals (8 cows/group) were collected at week 0, 8 and 16 by puncture biopsy after anaesthetizing the area with a subcutaneous lidocaine injection. Samples of one cow (GLY_LC_) were lost. The tissue samples were frozen immediately in liquid nitrogen and stored at -80°C until further processing. Blood samples were taken from the external jugular vein after milking in the morning at week 0, 4, 8, 12, 16.

### Feedstuff analyses

Feed samples dried at 60°C were analyzed for DM and chemical compositions [19] according to the standard methods of the VDLUFA [20]. Glyphosate concentrations in feed were measured by an accredited laboratory (Wessling GmbH, Altenberge, Germany) [19]. Data of chemical analyses together with the individually recorded feed intake was used to calculate individual intakes of nutrients, energy and GLY.

### Analytical procedures of blood samples

Blood samples were centrifuged for serum preparation (Heraeus Varifuge 3.0R Heraeus, Osterode, Germany; 2123 × g, 15°C, 15 min) and photometrically analyzed for serum concentrations of AST, glutamate dehydrogenase (GLDH), GGT, total bilirubin, cholesterol, total protein, albumin, triglyceride (TG) (Eurolyser^®^, Type VET CCA, Eurolyser Diagnostica GmbH, Salzburg, Austria), calcium and phosphorus (SPECORD 200 Plus, Analytik Jena AG, Jena, Germany). Concentrations of acetic acid, propionic acid, butyric acid and valeric acid in the blood plasma collected in week 0, 8 and 16 were analyzed by gas chromatography fitted with a flame ionization detector (GC-FID, Zebron ZB-1701, Phenomenex, Aschaffenburg, Germany) after derivatisation of short-chain fatty acids with 2-Chloroethyl chloroformate during sample preparation according to Kristensen [21].

### Histopathological analysis

Liver tissue for histopathological evaluation was sampled in week 0, 8 and 16. The biopsies underwent immersion fixation with 10% neutral buffered formalin, paraffin embedment, and hematoxylin and eosin (HE) staining of 4 μm sections. Microscopic analysis was performed by a pathologist certified by the American College of Veterinary Pathologists (ACVP). Standardized terms and criteria established for rodents [22] have been accordingly applied for classification and scoring of the hepatic lesions. To this end, the HE-stained sections were evaluated for lobular (S1A Fig) and portal inflammation (S1B Fig), intensity of infiltration with lymphocytes or plasma cells (S1C Fig), occurrence of hepatocellular apoptosis or necrosis (S1D Fig), fibrosis (S1E Fig), hemorrhage (S1F Fig), sinusoidal dilatation (S1G Fig), multinuclear hepatocytes (S1H Fig), glycogen (S1I Fig) and lipid storage (S1J Fig). Each parameter was assessed with 0 (= not present) or 1 (= present) and all scores were summarized as a cumulative overall liver histology score.

### RNA extraction

Total liver RNA from week 16 was isolated in RNAse-free water using the kit NucleoSpin^®^ RNA (Macherey-Nagel GmbH & Co. KG, Düren, Germany) according to the manufacturer’s protocol. Liver tissue was homogenized in lysis buffer using the SpeedMill Plus and innuSPEED Lysis Tube A (Analytik Jena AG, Jena, Germany) with two 30 second homogenization runs and a following shake-incubation for 5 min at room temperature. Quality and integrity measurement of isolated RNA was assessed using an Agilent 2100 Bioanalyzer (Agilent Technologies, Santa Clara, CA, USA). RNA integrity numbers of all samples were ≥ 7.5. Until further processing isolated RNA was frozen in liquid nitrogen and stored at -80°C.

### RNA sequencing and data processing

Two micrograms of total RNA and the TruSeq Stranded mRNA sample preparation kit were used for the preparation of sequencing libraries according to manufacturer’s protocol (Illumina, San Diego, CA, USA). The sequencing was conducted using multiplexed libraries and 2x 101 bp paired end reads on an Illumina HiSeq 2500 at the sequencing facility of the Institute of Genome Biology, Leibniz Institute for Farm Animal Biology (FBN), Dummerstorf, Germany. Before and after the data library processing steps sequence quality was checked with FastQC [23]. Raw reads were filtered and trimmed for minimal Phred scores of 20 and a minimum read length of 30 nt, while the terminal adapter sequence was removed using Trim Galore [24]. Filtered and quality checked reads were aligned to the bovine reference genome UMD3.1 (Ensembl release 93; [25]) using default parameters of Hisat2 version 2.1.0 [26]. Uniquely mapped reads were counted and assigned to gene features using HTSeq version 0.8.0 [27].

### Identification of differentially expressed genes

Genes with less than 20 assigned reads were excluded from further analysis based on the fact that genes with low counts have limited biological importance and statistical evidence [28]. The filtered data was used for normalization of gene counts and gene expression analysis. Differentially expressed genes (DEGs) were detected using DESeq2 version 1.20.0 [29] in RStudio version 1.1.456 [30] in R version 3.6.0 [31]. Normalization was conducted using the default shrinkage estimator with adaptive normal distribution [29]. Considering a p-value<0.05 and Benjamini Hochberg-adjusted p-value (p_adj_) <0.1 DEGs were identified comparing CON groups with GLY groups and HC with LC groups respectively. Comparisons between different biological conditions were analyzed by Venn analysis using the R package VennDiagram [32].

### Functional analysis and pathway enrichment

Functional analysis of all obtained DEGs determined by DESeq2 was performed using the Database for Annotation, Visualization and Integrated Discovery (DAVID) v.6.8 [33, 34] with lists of Ensembl-IDs of DEGs as input and default parameters for analysis [25]. Enrichment of Kyoto Encyclopedia of genes and Genomes (KEGG) pathways and molecular function (MF) was conducted. KEGG pathways and MFs were considered to be significantly enriched with thresholds of p<0.05 and a false discovery rate (FDR) of <10%. To confirm the acquired results of genes with annotated function and to refine these results with genes of unknown function, amino acid sequences were analyzed using BlastKOALA and the included database “family_eukaryotes” [35]. To retrieve these amino acid sequences, Ensembl-IDs of DEGs were converted to their corresponding NCBI protein accession numbers and respective amino acid sequences were collected using the NCBI Entrez API [36].

### cDNA synthesis and quantitative realtime PCR

For validation of the RNA sequencing (RNA-seq) approach, six CFP-responsive and five GLY-responsive genes of interest were chosen and subjected to a quantitative real time polymerase chain reaction (qRT-PCR). For this purpose, 800 ng RNA from the stock used for RNA-seq were used to synthesize cDNA by the qScript^™^ cDNA Synthesis Kit (Quanta Biosciences^™^, Inc, Gaithersburg, MD, USA) according to the manufacturer’s protocol. Until further analyses, cDNA was 1:10 diluted in H_2_O and stored at -20°C. Gene-specific primer pairs were designed using Primer-Blast [37], Beacon Designer (Free Edition Premier biosoft) and Primer3 version 4.0.0 [38, 39]. Primer selection and qRT-PCR conditions were conducted as described [40]. Expression and Cq values of genes were obtained by CFX Maestro^™^ 1.1 (Bio-Rad Laboratories, Inc, Hercules, CA, USA) using regression mode for Cq determination and utilizing actin beta (*ACTB*), ubiquitously expressed prefoldin like chaperone (*UXT*) and tyrosine 3-monooxygenase/tryptophan 5-monooxygenase activation protein zeta (*YWHAZ*) as reference genes showing a mean stability M value of 0.25. Information about selected primers is displayed in S1 Table. Normalized expression of genes of interest was calculated by using reference gene expression as normalization factor and taking primer specific efficiencies into consideration. Statistical analysis was performed on log transformed gene expression data.

### Statistical analyses

Histopathological scores and blood parameters were analyzed using the MIXED procedure with the restricted maximum likelihood model (reml) in SAS version 7.1 (SAS Institute Inc., Cary, North Carolina, USA). Week 0 was set as covariate in the class statement, while time (t; weeks of experiment), treatment (GLY; GLY or CON diet) and CFP (HC or LC diet) as well as their interactions (CFP*t, CFP*GLY, GLY*t, CFP*GLY*t) were applied as fixed factors. Time was treated as repeated measurement and specified within the subject cow. Variance component structure (vc) was used as covariance structure for all variables based on smallest Akaike information criterion compared to compound symmetry covariance structure (cs), autoregressive covariance structure (ar) and unstructured covariance (un) [41]. Unless otherwise stated, values are shown as Least Square means (LS means) and pooled standard error of the mean (PSEM). To analyze the qRT-PCR expression data, Kruskal-Wallis test was applied to log transformed expression values for gene comparisons that already showed differential expression in RNA-seq (Rstudio version 1.1.456). For calculation of Spearman’s correlation between RNA-seq data and qRT-PCR data, Rstudio (version 1.1.456) was used. Performance data of the 31 animals in week 16 were analyzed by ANOVA with treatment and CFP as fixed factors using the statistical software TIBCO Statistica 13.3 [42]. The MIXED procedure, ANOVA, Kruskal-wallis test and correlations were declared as highly significant when p<0.01 and significant when 0.01≤p≤0.05, while 0.05<p<0.1 were declared as trend. For linking the gene expression data with blood and performance parameters, sPLS was performed using RStudio version 1.1.456 [30] in R version 3.6.0 [31] with package mixOmics version 6.10.9 [43]. Blood parameter data were expanded with data of NEFA, glucose and BHB as well as performance data from this trial [19]. Gene count data were center log ratio (clr) transformed before analysis. Most suitable components were calculated using 20 repeats of 10-fold crossvalidation, while for correlations between gene expression and *in vivo* responses a robust Pearson’s-like -0.6<r<0.6 was chosen.

## Results

### Blood parameters

**Fig 1:**
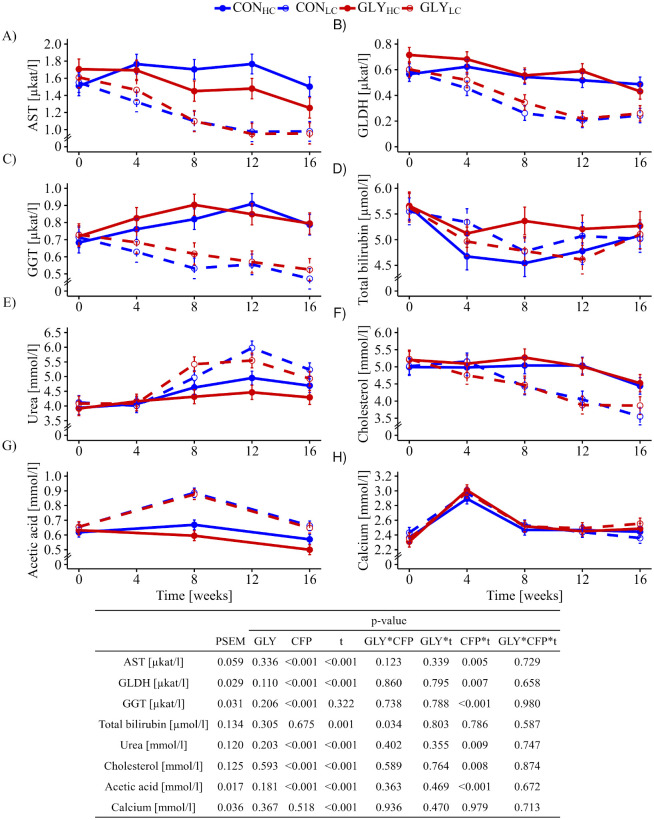
Influence of glyphosate residues and different concentrate feed proportions in diets of cows on biochemical blood parameters. Serum AST (A), GLDH (B), GGT (C), total bilirubin (D), urea (E), cholesterol (F), acetic acid (G) and calcium (H) of dairy cows fed with either a GLY-contaminated (GLY groups) or control (CON groups) diet consisting of high (HC, 60%) or low (LC, 30%) concentrate feed proportions were measured (CON_HC_, n = 16; CON_LC_, n = 16; GLY_HC_, n = 15; GLY_LC_, n = 14). Values are presented as LS means ± standard error of the mean. Parameters were analyzed with values from week 0 as covariate. PSEM = pooled standard error of the mean; GLY = glyphosate; CFP = concentrate feed proportions; t = experimental time; AST = aspartate aminotransferase; GGT = γ-glutamyltransferase; GLDH = glutamate dehydrogenase; CON = control; HC = high concentrate proportion in the diet; LC = low concentrate proportion in the diet.

Starting from comparable activities, AST (p_CFP*t_ = 0.005), GGT (p_CFP*t_<0.001) and GLDH (p_CFP*t_<0.007) of HC groups varied at higher levels over the course of the experiment in comparison with the LC groups (Fig 1A–1C) resulting in significant interactions between time and CFP. Total bilirubin levels were considered to be affected by time and in an interactive manner between GLY and CFP (p_GLY*CFP_ = 0.034, Fig 1D). The concentration of urea started to increase from week 4 onwards in LC groups while HC groups maintained their urea levels until the end of the experiment giving rise to significant interactions between CFP and time (p_CFP*t_ = 0.009, Fig 1E). Cholesterol levels remained constant until week 12 in HC groups and dropped slightly thereafter while a linear decrease was noticed in LC groups from week 4 irrespective of GLY exposure, which explained the significant interactions between time and CFP (p_CFP*t_ = 0.008, Fig 1F). Furthermore, higher acetic acid concentrations in LC groups compared to HC groups with a peak in week 8 were resulting in significant interactions between CFP and time (p_CFP*t_<0.001, Fig 1G). Propionic acid concentrations were mostly lower than the indicated limits of quantification (LOQ), while concentrations of butyric acid and valeric acid were even lower than the indicated limits of detection (LODs). Phosphorus levels varied inconsistently over time and partially in opposite directions giving rise to the significant interactions between time and CFP (p_CFP*t_<0.05, Table 1). TG levels decreased until week 8 in all groups, but at a lower level in HC groups before an increase was noticed in all groups (Table 1, p_CFP_ = 0.035, p_t_ = 0.003). Blood levels of albumin and total protein decreased significantly in all groups over the experimental time (p_CFP,t_<0.001, Table 1), while calcium levels remained stable except a peak in week 4 (p_t_<0.001, Fig 1H).

**Table 1:** Effects of GLY-contaminations and different CFP on blood metabolites.

		week of experiment								p-value				
	group	0	4	8	12	16	PSEM	GLY	CFP	t	GLY* CFP	GLY*t	CFP*t	GLY* CFP*t
Albumin[g/L]	CON_HC_	36.00	36.96 ^b^	33.57	35.92	33.66	0.526	0.835	0.265	**<0.001**	0.323	0.589	0.495	0.933
	CON_LC_	36.52	35.99	32.86	34.09	31.59 ^a^								
	GLY_HC_	36.13	36.40	33.98	34.43	32.33								
	GLY_LC_	37.34 ^b^	36.03	34.11	32.60	32.82								
Phosphorus [mmol/L]	CON_HC_	1.15	1.34	1.38	1.31	1.29	0.043	0.966	0.974	**<0.001**	0.180	0.797	**0.031**	0.374
	CON_LC_	1.26	1.22	1.32	1.23^a^	1.17								
	GLY_HC_	1.01	1.35	1.19	1.43	1.24								
	GLY_LC_	0.97 ^b^	1.39	1.38	1.35	1.38								
Total protein [g/L]	CON_HC_	70.64	73.48	65.57	71.79	66.72	1.280	0.402	0.181	**<0.001**	0.854	0.552	0.146	0.584
	CON_LC_	72.49	72.68	64.82	67.72	61.43								
	GLY_HC_	71.15	72.50	69.51	72.71	66.11								
	GLY_LC_	74.18	72.36	67.95	63.15	67.40								
Triglycerides [mmol/L]	CON_HC_	0.121	0.098	0.093	0.129	0.110	0.005	0.985	**0.035**	**0.003**	0.238	0.791	0.593	0.678
	CON_LC_	0.127	0.134	0.107	0.131	0.120								
	GLY_HC_	0.126	0.113	0.105	0.121	0.110								
	GLY_LC_	0.128	0.116	0.106	0.119	0.125								

Values are presented as LS means. Superscripted letters indicate statistically significant different groups. Parameters were analyzed with values from week 0 as covariate. PSEM = pooled standard error of the mean. GLY = glyphosate; CFP = concentrate feed proportions; t = experimental time; CON = control; HC = high concentrate proportion in the diet; LC = low concentrate proportion in the diet.

### Histopathology of the liver

**Fig 2:**
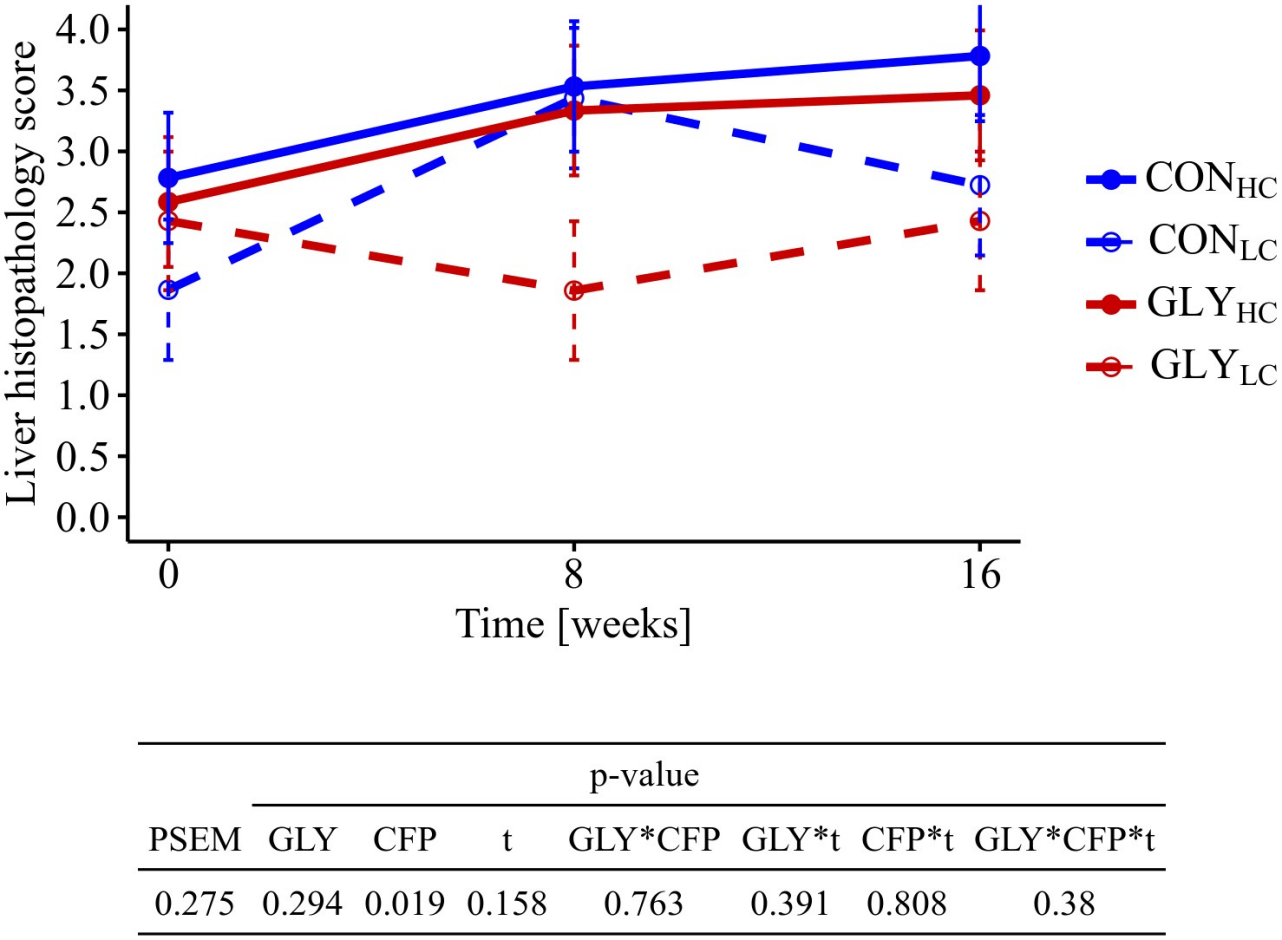
Influence of glyphosate residues and different concentrate feed proportions in diets of cows on liver histopathology. Cows were fed with either a GLY-contaminated (GLY groups) or a control (CON groups) diet consisting of high (HC, 60%) or low (LC, 30%) concentrate feed proportions (CON_HC_, n = 8; CON_LC_, n = 8; GLY_HC_, n = 8; GLY_LC_, n = 7). HE-stained liver sections were evaluated regarding the presence of ten different parameters and summarized in a cumulative overall liver histopathology score (0 = inconspicuous, 10 = conspicuous). Values are presented as LS means ± standard error of the mean. Parameters were analyzed with values from week 0 as covariate. PSEM = pooled standard error of the mean; CON = control; GLY = glyphosate; CFP = concentrate feed proportions; t = experimental time; CON = control; HC = high concentrate proportion in the diet; LC = low concentrate proportion in the diet.

The cumulative liver histopathology scores were significantly higher in HC groups than in LC groups, while no significant GLY effects were observable (p_CFP_<0.019, Fig 2). Having a closer look at the single parameters forming the liver histopathology score, occurrence of hepatocellular apoptosis or necrosis, portal inflammation, intensity of infiltration with lymphocytes or plasma cells, multinuclear hepatocytes cells as well as sinusoidal dilation were the main reasons for elevated scores in HC groups.

### RNA sequencing analysis

**Fig 3:**
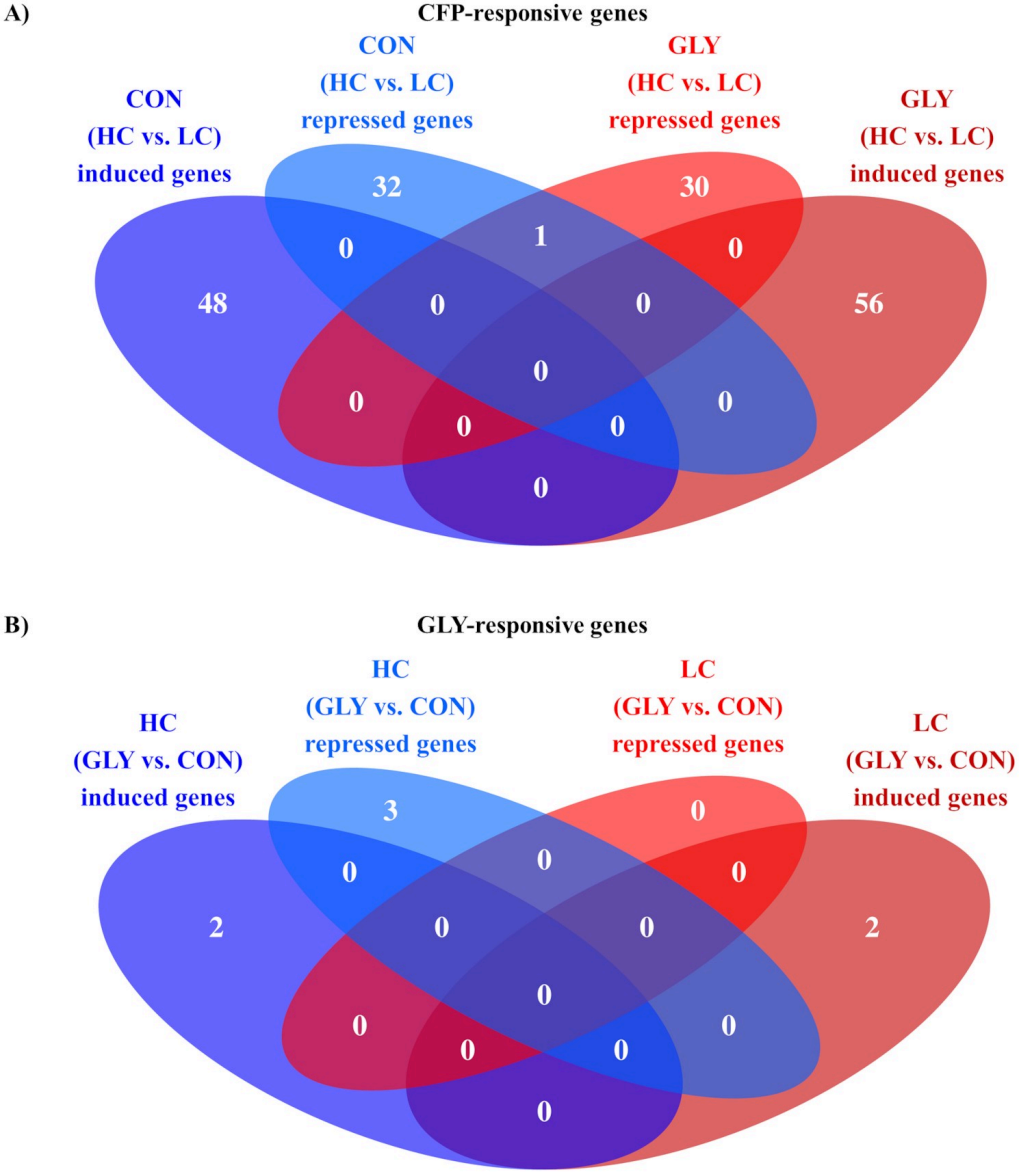
Influence of glyphosate residues and different concentrate feed proportions in diets of dairy cows on liver transcriptome. Cows fed with either GLY-contaminated (GLY groups) or control (CON groups) diets containing high (HC) or low (LC) concentrate feed proportions in their rations. The Venn diagrams show the differentially expressed genes (p<0.05, p_adj_<0.1) after comparing the groups with varying CFP (HC vs. LC, A) or treatment (GLY vs. CON, B). CON = control; GLY = glyphosate; CFP = concentrate feed proportions; HC = high concentrate feed proportion in the diet; LC = low concentrate feed proportion in the diet.

On average 32,940,447 reads were generated per sample. 63.3% of the reads could uniquely mapped to genes in the *Bos taurus* genome, while 15.5% of the reads uniquely mapped to intergenic regions. The remaining reads could either not be uniquely mapped or showed too low quality. The RNA-seq analysis displayed a total of 167 DEGs (p<0.05, p_adj_<0.1) upon varying CFP (HC vs. LC) and seven DEGs upon GLY-contaminations (GLY vs. CON, Fig 3). Of all CFP-responsive DEGs, 81 were found in CON groups and 87 in GLY groups (Fig 3A). Furthermore, 104 CFP-responsive genes (48 in CON_HC_, 56 in GLY_HC_) showed a higher transcript abundance in comparison to respective LC groups, while 63 genes (33 in CON_HC_, 31 in GLY_HC_) were decreased in their expression (Fig 3A). Besides an overlap of one gene, all repressed CFP-responsive genes were unique to CON and GLY groups. On the other side, seven genes were differentially expressed upon dietary GLY exposure (GLY vs. CON), while five DEGs were found in HC groups and two DEGs in LC groups (Fig 3B). Four of these genes (two in GLY_HC_, two in GLY_LC_) showed an increased expression upon dietary GLY-uptake, while three genes (three in GLY_HC_, zero in GLY_LC_) were repressed (Fig 3B). Detailed information about DEGs including IDs, name, description and statistical information are shown in S2 and S3 Tables. A general overview of transcriptome alterations in form of DEGs caused by GLY-contaminations or different CFP in dairy cows’ diets is shown in Fig 3.

### Functional characterization of CFP- and GLY-responsive genes

According to the DAVID database, 158 of all 167 CFP responsive genes could be assigned to gene functions in the *Bos taurus* genome using DAVID’s default settings. 98 of these assigned genes could be mapped to ten different KEGG pathways (S4 Table). Applying a significance threshold to the KEGG pathways (p<0.05, FDR<10%), 21 genes mapped to four KEGG pathways remained.

**Fig 4:**
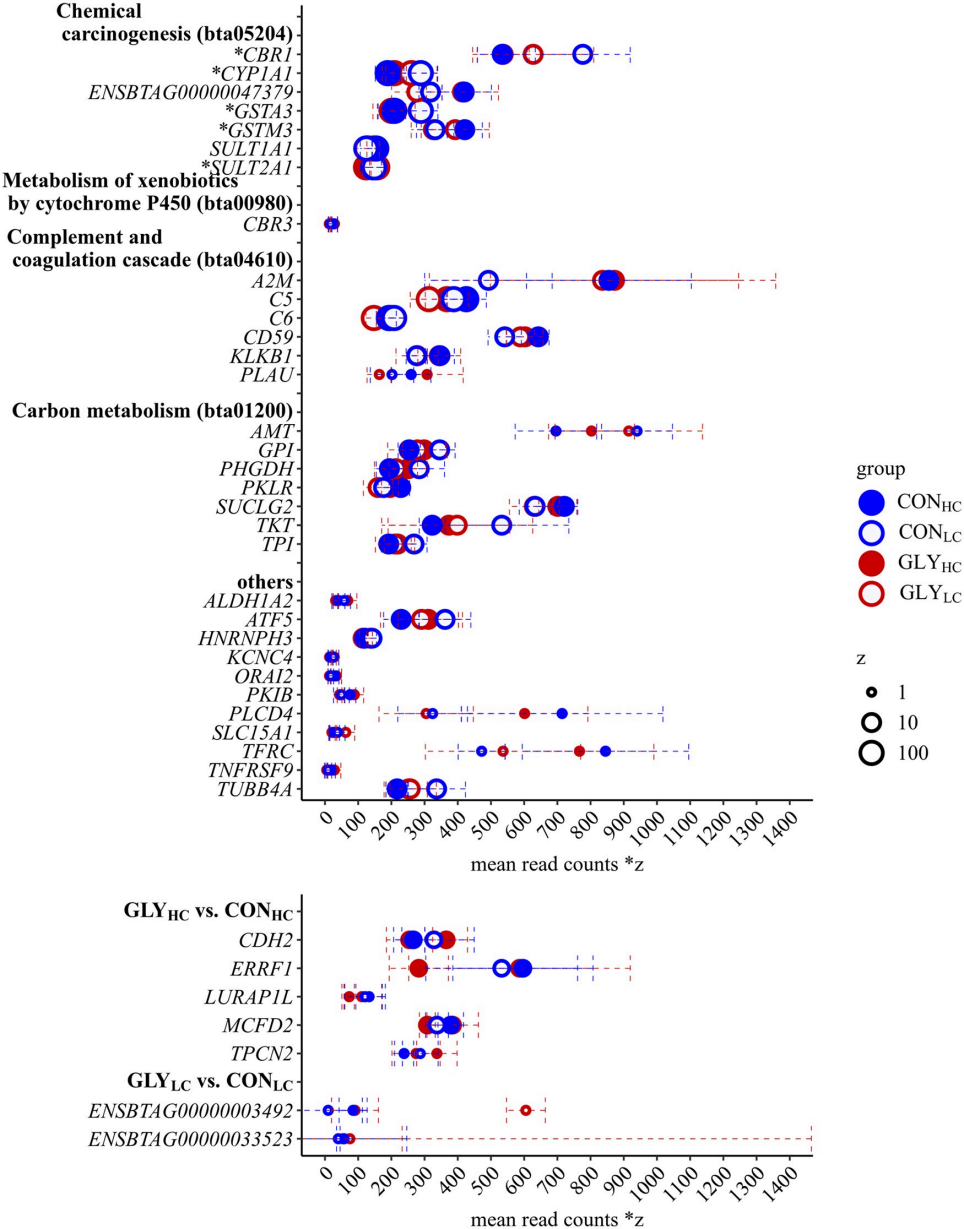
Differentially expressed genes with mean normalized read counts in respective feeding groups according to RNA-sequencing. Shown are mean normalized read counts (dots) and standard deviation (error bar) of KEGG pathway enriched DEGs and further non pathway enriched DEGs of particular interest in respective feeding groups (CON_HC_, CON_LC_, GLY_HC_ and GLY_LC_) upon different CFP (A) as well as all DEGs upon GLY-contaminations (B) in dairy cows’ rations. The size of dots represents the expression level, while Factor z was used for scaling. *DEGs were also enriched in “metabolism of xenobiotics by cytochrome P450”; CON = control; GLY = glyphosate; CFP = concentrate feed proportions; HC = high concentrate feed proportion in the diet; LC = low concentrate feed proportion in the diet.

In detail, according to DAVID, both “chemical carcinogenesis” and “carbon metabolism” were represented by seven assigned DEGs, while “metabolism of xenobiotics by cytochrome P450” and the “complement and coagulation cascades” were represented by six assigned DEGs, respectively (Table 2). The assignment of DEGs to KEGG pathways in DAVID was validated and expanded by the analysis in BlastKOALA. Genes which were assigned to “chemical carcinogenesis” according to DAVID, were identified as *CBR1*, *CYP1A1*, *GSTA3*, *GSTM3*, *SULT1A1*, *SULT2A1* and the uncharacterized gene ENSBTAG00000047379 (S2 Fig). The DEGs *CBR1*, *CYP1A1*, *GSTA3*, *GSTM3* and *SULT2A* were also identified in the “metabolism of xenobiotics by cytochrome P450”. Furthermore, *CBR3* was identified as DEG within this pathway (S3 Fig). According to BlastKOALA, alcohol dehydrogenase 1/7 (*ALDH1_7)* was additionally enriching the pathway “chemical carcinogenesis” and “metabolism of xenobiotics by cytochrome P450”, although this gene could not be found as CFP-responsive within the RNA-seq data set. All six DEGs enriching “complement and coagulation cascades” were induced while expression of *PLAU* differed most (lfc = 0.52). *A2M*, *C5*, *C6*, *CD59* and *KLKB* were also enriched (S4 Fig). According to BlastKOALA, the complement factor H (*CFH7*, lfc = 0.48) expanded the DEGs in “complement and coagulation cascades”, while *A2M* was only identified in DAVID. As DEGs related to “carbon metabolism” *AMT*, *GPI*, *PHGDH*, *PKLR*, *SUCLG2*, *TKT* and *TPI* were identified (S5 Fig). In HC groups, the top five downregulated genes not enriching KEGG pathways were solute carrier family 15 member 1 (*SLC15A1*, lfc = -0.62), aldehyde dehydrogenase 1 family member A2 (*ALDH1A2*, lfc = -0.57), potassium voltage-gated channel subfamily C member 4 (*KCNC4*, lfc = -0.53), activating transcription factor 5 (*ATF5*, lfc = -0.42) and tubulin beta 4A class IVa (*TUBB4A*, lfc = -0.41). Heterogeneous nuclear ribonucleoprotein H3 (*HNRNPH3*, lfc = -0.22) was the only gene showing a downregulation in both HC groups. cAMP-dependent protein kinase inhibitor beta (*PKIB*, lfc = 0.56), ORAI calcium release-activated calcium modulator 2 (*ORAI2*, lfc = 0.56), transferrin receptor (*TFRC*, lfc = 0.52), phospholipase C delta 4 (*PLCD4*, lfc = 0.49) and tumor necrosis factor receptor superfamily member 9 (*TNFRSF9*, lfc = 0.48) were the top five genes that were induced in HC groups but not enriched in KEGG pathways. In contrast to the CFP-responsive genes, the seven DEGs upon dietary GLY uptake were not sufficient for functional characterization using DAVID. Accordingly, no KEGG pathways were considered to be significantly enriched by GLY-responsive genes. In detail, the DEGs in GLY_HC_ compared to CON_HC_ were cadherin 2 (*CDH2*, lfc = 0.32), ERBB receptor feedback inhibitor 1 (*ERRFI1*, lfc = -0.36), leucine rich adaptor protein 1 like (LURAP1L, lfc = -0.38), multiple coagulation factor deficiency 2 (MCFD2, lfc = -0.25) and two pore segment channel 2 (*TPCN2*, lfc = 0.34). The two GLY-responsive genes (ENSBTAG00000033523, ENSBTAG00000003492) in GLY_LC_ compared to CON_LC_ encoded uncharacterized proteins and were, therefore, excluded from further analysis. Further characterization of the seven detected GLY-responsive genes outside of KEGG pathways revealed that *CDH2* encodes a calcium-dependent transmembrane protein mediating cell-cell adhesion [44], *ERRFI1* encodes a negative regulator of epidermal growth factor receptor [45, 46] and *LURAP1L* is predicted to be an adaptor protein that contains two leucine-repeats in tandem [47]. *TPCN2* encodes a nicotinic acid adenine dinucleotide phosphate-dependent Ca^2+^-release channel [48] and *MCFD2* encodes a part of coagulation factor transporting complex [49]. Of the GLY-responsive genes two genes (*CDH2*, *MCFD2*) were aligned to “GO:0005509~calcium ion binding,” within GOTERM MF. The mean normalized read counts of all pathway enriching CFP-responsive genes as well as possibly GLY-responsive genes are shown in Fig 4.

**Table 2:** Functional clustering of CFP-responsive genes by using DAVID.

			KEGG Pathway	Chemical carcinogenesis (bta05204)	Metabolism of xenobiotics by cytochrome P450 (bta00980)	Complement and coagulation cascades (bta04610)	Carbon metabolism (bta01200)
		p-value		<0.001	<0.001	0.002	0.002
			FDR	0.312	1.091	2.991	3.183
Gene name	symbol	lfc^$^					
carbonyl reductase 1	*CBR1*	-0.40		+	+		
cytochrome P450, subfamily I (aromatic compound-inducible), polypeptide 1	*CYP1A1*	-0.43		+	+		
ENSBTAG00000047379	-	0.30		+			
glutathione S-transferase alpha 3	*GSTA3*	-0.32		+	+		
glutathione S-transferase mu 3	*GSTM3*	0.27		+	+		
sulfotransferase family, cytosolic, 1A, phenol-preferring, member 1	*SULT1A1*	0.26		+			
sulfotransferase family, cytosolic, 2A, dehydroepiandrosterone (DHEA)-preferring, member 1	*SULT2A1*	-0.31		+	+		
carbonyl reductase 3	*CBR3*	0.50			+		
alpha-2-macroglobulin	*A2M*	0.35				+	
complement C5	*C5*	0.28				+	
complement C6	*C6*	0.30				+	
CD59 molecule	*CD59*	0.23				+	
kallikrein B1	*KLKB1*	0.27				+	
plasminogen activator urokinase	*PLAU*	0.52				+	
aminomethyltransferase	*AMT*	-0.29					+
glucose-6-phosphate isomerase	*GPI*	-0.36					+
phosphoglycerate dehydrogenase	*PHGDH*	-0.34					+
pyruvate kinase	*PKLR*	0.32					+
succinate-CoA ligase GDP-forming beta subunit	*SUCLG2*	0.18					+
transketolase	*TKT*	-0.40					+
triosephosphate isomerase	*TPI*	-0.40					+

^$^Values generated from RNA-sequencing; FDR = false discovery rate; lfc = log2 fold change.

To get further insights and to link gene expression results with *in vivo* responses, expression of DEGs was compared to performance data (S1 Table) and clinical chemistry data using a PLS analysis. Correlations (-0.6>r>0.6) of 29 DEGs with levels of blood parameters (albumin, glucose, total protein, NEFA, AST and GLDH) and performance parameters (DMI, milk yield and energy-corrected milk yield) was observed (S6 Fig). All of the 29 DEGs were CFP-responsive, whereas correlations of GLY-responsive DEGs were not observed.

### qRT-PCR

Validity of RNA-seq results was confirmed by qRT-PCR. Due to low changes in gene expression, the genes with the strongest changes in expression for each CFP-responsive enriched KEGG pathway (*CYP1A1*, *PLAU*, *TKT*, *TPI*) as well as additional genes of interest (*ATF5*, *TNFRSF9*) were chosen for qRT-PCR. Additionally, five GLY-responsive genes from the RNA-seq approach (*CDH2*, *ERRFI1*, *LURAP1L*, *MCFD2*, *TPCN2*) were chosen for validation. All genes were consistent between methodologies in direction and magnitude of changes in their expression (Table 3). In qRT-PCR, statistically significant effects of GLY on gene expression were detected for *CDH2* (p = 0.012) and *ERRFI* (p = 0.021) comparing GLY_HC_ and CON_HC_, while changes in *LURAP1L* expression showed a trend (p = 0.074). Expression of *MCFD2* (p = 0.208) and *TPCN2* (p = 0.141) showed no significant differences when comparing GLY_HC_ to CON_HC_. Within the CFP-responsive genes analyzed by qRT-PCR *TNFRSF9* (p = 0.026) showed a significant effect of CFP comparing GLY_HC_ and GLY_LC_. For *ATF5* (p = 0.528), *CYP1A1* (p = 0.141), *PLAU* (p = 0.151), *TKT* (p = 0.345) and *TPI* (p = 0.294) no significant effects were observable when comparing corresponding HC group and LC group. However, Spearman correlation coefficients between normalized read counts of the RNA-seq approach and Cq values of qRT-PCR were significant for *ATF5*, *PLAU*, *TKT*, *TPI*, *TNFRSF9*, *CDH2*, *ERRFI*, *LURAP1L* while a trend was noticed for *TPCN2* (Table 3). Correlation coefficients were negative since an increase in read counts was associated with a decrease in Cq values.

**Table 3:** Selected relevant differentially expressed genes analyzed in RNA-seq and validation by qRT-PCR.

Symbol	gene name	treatment group	control group	Log2 fold change (treatment/control)		Spearman correlation (r)
				RNA-seq	qRT-PCR	
*ATF5*	activating transcription factor 5	CON_HC_	CON_LC_	-0.43^###^	-0.31	-0.34^###^
*CYP1A1*	cytochrome P450, subfamily I (aromatic compound-inducible), polypeptide 1	CON_HC_	CON_LC_	-0.43^###^	-0.36	-0.24
*PLAU*	plasminogen activator, urokinase	GLY_HC_	GLY_LC_	0.52^###^	0.45	-0.59^#^
*TKT*	transketolase	CON_HC_	CON_LC_	-0.4^###^	-0.38	-0.64^#^
*TNFRSF9*	tumor necrosis factor receptor superfamily member 9	GLY_HC_	GLY_LC_	0.48^###^	1.69^##^	-0.73^#^
*TPI*	triosephosphate isomerase 1	CON_HC_	CON_LC_	-0.40^###^	-0.33	-0.39^##^
*CDH2*	cadherin 2	GLY_HC_	CON_HC_	0.32^###^	0.50^##^	-0.64^#^
*ERRFI*	ERBB receptor feedback inhibitor 1	GLY_HC_	CON_HC_	-0.36^###^	-0.90^##^	-0.56^#^
*LURAP1L*	leucine rich adaptor protein 1 like	GLY_HC_	CON_HC_	-0.38^###^	-0.42^###^	-0.45^##^
*MCFD2*	multiple coagulation factor deficiency 2	GLY_HC_	CON_HC_	-0.25^###^	-0.19	-0.08
*TPCN2*	two pore segment channel 2	GLY_HC_	CON_HC_	0.34^###^	0.39	-0.49^#^

^#^ p<0.01,

^##^ p<0.05,

^###^ 0.05<p<0.1;

GLY = glyphosate; CON = control; HC = high concentrate feed proportion in the diet; LC = low concentrate feed proportion in the diet.

## Discussion

Since GLY is one of the most used non-selective herbicides in agriculture worldwide, GLY residues can be found in dairy cow rations [5]. According to von Soosten et al. [5] and Krüger et al. [50], cows are frequently exposed to GLY and, therefore, have to cope with these xenobiotic residues. The liver as a primary target of xenobiotics like GLY, is frequently analyzed in studies in cell cultures [13] or studies in laboratory animals [10–12]. However, there is a lack of *in vivo* trials examining effects of GLY on livestock under exposure conditions that could arise from agricultural practice. Besides GLY exposure, these conditions include varying concentrate proportions in the rations which might have marked effects both on hepatic nutrient and xenobiotic metabolism. Therefore, this study considered maximum exposure conditions for the GLY formulation Round Record^®^ with GLY as active substance according to legal applications. It examined the GLY effects upon different CFP on the health of lactating dairy cows with particular emphasis on liver.

The daily GLY uptake in this study was 13x higher than maximal GLY uptake of 6.67 mg/d in dairy cows [5]. Translated, this lead to a cows’ average daily GLY exposure of 1.20 (CON_LC_), 1.11 (CON_HC_), 112.6 (GLY_LC_) and 132.8 μg/kg body weight (GLY_HC_), which, in GLY groups, is even approximately 30x higher than the average background exposure of dairy kept at the experimental station in Braunschweig [19], while a higher DM intake led to higher GLY exposure in GLY_HC_ [19]. Since this study was conducted under common practical conditions in agriculture, the cows in CON groups were minimally exposed to GLY residues as mentioned above. These GLY-contaminations of the CON rations might be resulting from background contaminations of feedstuffs regularly occurring in dairy cows’ feed [5].

Our results demonstrated no adverse effects of the tested GLY formulation on analyzed parameters such as enzyme activities of AST, GGT and GLDH as indicators of liver integrity [51, 52] and biochemical blood parameter levels associated to hepatic function including synthesis capacity (e.g. albumin, cholesterol and total bilirubin), liver histology and hepatic gene expression, whereas varying CFP and time affected some of these parameters. In contrast to our findings of missing GLY effects, Benedetti et al. [11] reported an up to one and a half fold increase of AST activity in rats fed with 48.7 and 487 mg/kg body weight GLY every two days for a period of 75 days. Jasper et al. [12] detected a significant increase in AST and GGT activity in mice treated with 50 and 500 mg/kg body weight for 15 days. Additionally, Krüger et al. [50] supposed an increase of GLDH levels in Danish cows to be associated with a not further specified GLY exposure. Assessing the discussed GLY effects on laboratory animals it needs to be stressed that exposure levels were manifold higher than that applied in the current controlled feeding study with cows.

In contrast to GLY exposure, energy supply (net energy (NE) intake: 145 MJ NE requirement for lactation (NE_L_)/d (CON_HC),_ 144 MJ NE_L_/d (GLY_HC_), 112 MJ NE_L_/d (CON_LC_), 112 MJ NE_L_/d (GLY_LC_), [19]) resulted in marked differences between HC and LC groups. Generally, feeding of the HC diets appeared to be related to higher AST, GGT and GLDH activities relative to LC groups which might largely reflect the increased hepatic nutrient turnover driven by the stimulated DM intake and thus nutrient intake. Supporting this assumption, the higher blood cholesterol levels in cows fed the HC diets can be considered as an indicator of the enhanced hepatic nutrient turnover.

Serum bilirubin and albumin levels are often used as markers for hepatocyte function [51]. Owagboriaye et al. [53] reported a higher level of plasma bilirubin as well as a reduction of albumin in rats treated with Roundup Original^®^ for a period of 12 weeks (3.6–248.4 mg/kg body weight) and concluded a putative liver dysfunction. In the present trial, GLY showed no effect on serum albumin levels, whereas serum bilirubin was temporarily elevated in week 8 solely in group GLY_HC_. The reasons remain unclear, since no other GLY effects on liver-related parameters were observed as described in this work and by Schnabel et al. [18].

In contrast to our findings that serum urea concentrations were not responsive to GLY, Dedeke et al. [54] observed a significant increase of blood urea levels in rats treated with 50.4–248.4 mg/kg body weight. In contrast to rats, the amounts of urea in blood and milk of ruminants result from ruminal protein catabolism and rumino-hepatic N-cycling and are consequently depending on diet composition in general and particularly on energy supply available for microbial protein synthesis [55, 56]. Rations of LC groups contained higher amounts of crude protein but lower energy levels in conjunction with higher neutral detergent fibre (NDF) levels [19] which could have led to higher concentration of blood urea levels in LC groups than in HC groups over time following an adaption period of four weeks. Fermentation of structural carbohydrates which are represented by the NDF fraction and which are typical for LC diets results in higher ruminal acetate and lower propionate levels compared to the fermentation of starch [57, 58]. Consequently, this fermentation pattern resulted in higher systemic absorption of acetate in the blood.

Contrary to elevated cholesterol levels in the HC groups, the TG concentrations in peripheral blood declined in these groups. This might reflect a lower hepatic TG synthesis due to lower ruminal acetate supply as precursor for fatty acid synthesis [59].

However, missing GLY effects are not in line with [9], who reported an increase in serum TG levels in rats orally exposed to 4 ng/kg body weight GLY for two years. According to Fu et al. [60] GLY can lead to changes in lipid metabolism and fat deposition in the liver. They fed pigs with 10, 20 and 40 mg GLY/kg diet for 35 days. Histopathological evaluation revealed, for instance, increasing lipid granules, high degree of fibrosis or necrosis of hepatocytes with increasing GLY concentration in the diets [60]. However, neither an increase of serum TG levels nor any changes in liver histopathology after GLY exposure for 16 weeks were observed in the present study. In contrast to our findings, other authors reported liver abnormalities like hepatic congestions, macroscopic and microscopic necrotic foci [10], changes in connective tissue and collagen deposition [11] as well as nucleolar disruption in hepatocytes [9] in GLY-treated rats. The observed histopathological alterations in the present study only occurred upon different CFP in the diets. They were weak compared to a maximal score of 10 (maximal mean score: CON_HC (week 16)_ 3.78). An increased amount of hepatocellular apoptosis or necrosis were the major drivers for the slightly increased scoring in HC groups. This is in line with the observed higher AST, GGT and GLDH activities in the HC groups relative to the LC groups [61]. Furthermore, sinusoidal dilations, portal inflammation, presence of lymphocytes and plasma cells and multinuclear hepatocytes played a role in the liver score. In this study, slightly higher liver histopathology scores in HC groups could indicate generally higher metabolic liver activities as discussed above.

Varying CFP in the diets led to 167 DEGs in gene expression analysis, while seven genes were GLY-responsive. Of the CFP-dependent DEGs 21 were enriched in four biological pathways such as “metabolism of xenobiotics by cytochrome P450”, a pathway responsible for the degradation of xenobiotics [62–64] and “chemical carcinogenesis” which is a multistep process involved in chemically induced cancer development [65]. On the one hand, these pathway enrichments are likely false positive enrichments, since the assigned DEGs are randomly distributed within these overlapping pathways, while other genes within these pathways did not show CFP responsiveness. Furthermore, mentioned DEGs take part in additional metabolic processes like lipid metabolism (*CBR1*, *CBR3*, *CYP1A1)* [62–64], the sulfation of bile acids in the liver (*SULT2A1*; [66]) or steroid hormone biosynthesis (*CYP1A1*) [62–64]. Due to an increased hepatic nutrient turnover in HC groups, as discussed above, expression of mentioned genes might be also regulated by not further specified endogenous substrates. On the other hand, effects of varying dietary compositions on the expression of drug-metabolizing enzymes in the liver were reported in mice [17].

An induction of the “complement and coagulation cascades” which is a non-specific defense mechanism against pathogens [62–64] and links inflammatory response and coagulation [67] suggested an immune response. High concentrate diets were reported to lead to elevated LPS concentrations in the portal and hepatic vein in cows [61]. This consequently leads to enhanced expression of hepatic immunological relevant genes [68]. The enzyme PLAU which is involved in plasminogen conversion [69] and part of the complement and coagulation system was reported to be induced in mammary tissue of postpartal cows after intramammary LPS injection [70].

Finally, the pathway “carbon metabolism” consists of carbon utilizing pathways like glycolysis, the pentose phosphate pathway or the citrate cycle (KEGG). In our study, five genes (*AMT*, *GPI*, *PHGDH*, *TKT* and *TPI*) related to “carbon metabolism” were repressed upon higher concentrate feed proportions, while two genes were induced (*SUCLG2* and *PKLR*). Reasons for repression of glycolysis- and pentose phosphate-related genes *GPI*, *TKT* and *TPI* might be a reduction of energy generation by glycolysis and an enhancement of glycogen synthesis as a storage form of glucose in the liver [71]. This might be explained by higher energy levels in the diets of HC groups leading to an excess of glucose [19] which is not further required for energy generation and consequently stored as glycogen [71]. Furthermore, a reduced activity of the pentose phosphate pathway in the HC groups might further support the view of a reduced hepatic fatty acid synthesis capacity due to a lower NADPH availability which, in turn, reduces TG synthesis and peripheral export. Last but not least, only CFP-responsive DEGs occurred correlated (-0.6>r>0.6) with performance and blood data in PLS analysis, while GLY intake and GLY-responsive genes did not correlate with these parameters.

According to von Soosten et al. [5] consumed GLY is mainly excreted by urine (61 ± 11%) and feces (8 ± 3%). Missing GLY amounts are potentially degraded by ruminal microbiota or absorbed via the ruminal epithelium [5]. This absorption could be realized via the LAT1/LAT2 transporter system, since GLY is a glycine analogue [72]. However, GLY absorption capacity for the ruminal epithelium is low [5]. Since their balance studies were carried out within relatively constant energy levels in the diet (30–45% CFP based on DM) [5], influences on the absorption capacity of GLY in the context of high CFP in the diet and resulting changed ruminal microbiome and fermentation characteristics cannot be excluded [73]. Nevertheless, Fu et al. [60] postulated GLY-metabolizing properties of the liver as they detected GLY residues in liver of weaning pigs after GLY intake. Other authors [9] reported hepatic gene expression alterations for more than 4000 genes in rats following a chronic GLY-exposure of 4 ng/kg body weight. However, only p-values were used as significance threshold for DEG determination in this study and liver samples were obtained from the study of Séralini et al. [10], which was retracted in 2012 and republished in 2014. It should be noted that the study is highly debated in the scientific community as reviewed by Resnik [74]. Accordingly, genes with altered expression in the liver upon GLY exposure presented in [9] have to be considered with caution. These genes were associated with, for instance, metabolic stress related pathways or apoptosis. In contrast to the drastic hepatic gene expression alterations in [9], only seven GLY-responsive genes were observed in the present study. These changes in expression can even be attributed to a false-positive detection, as this number of genes is low in comparison with the underlying genome size. Additionally, fold-changes for these genes were weak with a maximum increase of 1.4fold and read counts were low with an average of 367 in GLY_HC_ (*TPCN2*) or a maximum decrease of 1.8fold with an average of 83 read counts in GLY_HC_ (*LURAP1L*). This also explains that the detected expression changes in GLY_HC_ by qRT-PCR were only statistically validated for *CDH2*, *ERFFI* and *TPCN2*. Seven DEGs were not enough for enriching KEGG signaling pathways, since the statistic’s power for enrichment is very limited in small gene lists [33]. According to DAVID and further characterization, three GLY-responsive genes (*CDH2*, *MCFD2* and *TPCN2*) are related to calcium binding. Since GLY is considered to be a chelator for metal ions like calcium [75], GLY and calcium levels in the blood were measured to analyze potential chelating behavior of GLY. Calcium concentrations showed no significant GLY effect and GLY levels in the blood were lower than the detection limit of 0.59 μM [18]. To improve the results, advanced techniques in detection of GLY concentration in blood were used for more precise measurement of these. Mean calcium levels (2539 μM) and mean GLY concentration (0.017 μM) in blood of randomly selected cows from both GLY groups in week 16 of the trial were used for a calculation of the potential formation of 1:1 calcium/GLY complexes [76, 77]. Calculations resulted in a mean of ~146,000fold calcium excess in GLY groups. Even if GLY affected calcium levels, which is unlikely as described in Buffler et al. [78], this would not be enough to explain changes in expression of calcium related genes in the liver, in the case that GLY levels in blood and hepatocytes would be comparable, what is still unknown. Additionally, studies reported that calcium ions were able to inhibit GLY properties *in vitro* [77] which would rather suggest a GLY neutralization than a negative effect of calcium chelation. Consequently, interactions between GLY and calcium in the blood of dairy cows were considered to be unlikely or non-relevant in our study. Ignoring that GLY-responsive genes could be false-positive genes and assuming minimal changes in gene expression in the present study, the biological relevance of this is questionable, since the tested GLY formulation showed no adverse effects on liver histopathology, biochemical parameters as well as general animal health characteristics [19] and hematological parameters [18] upon practical maximum GLY exposure conditions. Finally, it should be pointed out, that Roundup Record^®^ is a formulation and contained other ingredients, especially surfactants, besides GLY, which might have possible toxic effects. Previous studies likely used GLY formulations with different surfactants, which had been shown to have more toxic potential than the surfactant in Roundup Record^®^ [79] and were banned in the EU. This has to be taken into consideration when comparing results of older studies to this study, where ethoxylated fatty amidoamine was used as surfactant in the GLY formulation according to European regulations.

## Conclusion

The present study aimed to represent real-life worst-case conditions of GLY-contaminations in dairy cows’ rations. Our findings showed, that the tested GLY formulation did not induce adverse effects on biochemical blood parameters, liver histopathology as well as on hepatic gene expression in lactating dairy cows, whereas different concentrate feed proportions in the diet, time or an interaction between them affected all mentioned parameters. Thus, it can be concluded, that upon conditions applied in our study, no adverse effects of the tested GLY formulation occur on dairy cows regarding the analyzed parameters.

## Supporting information

S1 Fig Histopathological analysis of Hematoxylin and Eosin (HE) stained liver tissue. For classification and scoring of the hepatic lesions HE-stained sections were evaluated for lobular (A) and portal inflammation (B), intensity of infiltration with lymphocytes or plasma cells (C), occurrence of hepatocellular apoptosis or necrosis (D), fibrosis (E), hemorrhage (F), sinusoidal dilatation (G), multinuclear hepatocytes (H), glycogen (I) and lipid storage (J). Shown are examples where respective parameters were assessed with 1 (= present). All scores were summarized as a cumulative overall liver histology score.
(TIF)

S2 Fig Assignment of concentrate feed proportion responsive genes in the liver to KEGG pathway “Chemical carcinogenesis (bta05204)”. According to DAVID “Chemical carcinogenesis” was enriched with seven differentially expressed genes. Genes were induced (green) or repressed (brown) in liver of cows fed with high concentrate feed proportions compared to those receiving low concentrate feed proportions in their ration for 16 weeks.
(TIF)

S3 Fig Assignment of concentrate feed proportion responsive genes in the liver to KEGG pathway “Metabolism of xenobiotics by cytochrome P450 (bta00980)”. According to DAVID “Metabolism of xenobiotics by cytochrome P450” was enriched with seven differentially expressed genes. Genes were induced (green) or repressed (brown) in liver of cows fed with high concentrate feed proportions compared to those receiving low concentrate feed proportions in their ration for 16 weeks.
(TIF)

S4 Fig Assignment of concentrate feed proportion responsive genes in the liver to KEGG pathway “Complement and coagulation cascades (bta04610)”. According to DAVID “Complement and coagulation cascades” was enriched with seven differentially expressed genes. Genes were induced (green) or repressed (brown) in liver of cows fed with high concentrate feed proportions compared to those receiving low concentrate feed proportions in their ration for 16 weeks.
(TIF)

S5 Fig Assignment of concentrate feed proportion responsive genes in the liver to KEGG pathway “Carbon metabolism (bta01200)”. According to DAVID “Carbon metabolism” was enriched with seven differentially expressed genes. Genes were induced (green) or repressed (brown) in liver of cows fed with high concentrate feed proportions compared to those receiving low concentrate feed proportions in their ration for 16 weeks.
(TIF)

S6 Fig PLS of differentially expressed genes in the liver with performance and biochemical blood parameters. Clustered image map of PLS analysis comparing in vivo responses and gene expression in the liver of cows fed with or without glyphosate-contaminated rations as well as with different concentrate feed proportions for 16 weeks. ADIPOR2 = adiponectin receptor 2; ALDH1L2 = aldehyde dehydrogenase 1 family member L2; AST = aspartate aminotransferase; BLVRB = biliverdin reductase B; CLDN4 = claudin 4; CORO1C = coronin 1C; CPM = carboxypeptidase M; CRAT = carnitine O-acetyltransferase; CTSC = cathepsin C; CTTN = cortactin; DMI = dry matter intake; ECM = energy-corrected milk yield; EPS15 = epidermal growth factor receptor pathway substrate 15; FAM102A = family with sequence similarity 102 member A; GLDH = glutamate dehydrogenase; GSTA3 = glutathione S-transferase alpha 3; LYPD1 = LY6/PLAUR domain containing 1; NEFA = non-esterified fatty acids; NQO1 = NAD(P)H quinone dehydrogenase 1; NR2F1 = nuclear receptor subfamily 2 group F member 1; ORAI = ORAI calcium release-activated calcium modulator 2; PKIB = protein kinase (cAMP-dependent, catalytic) inhibitor beta; RASGEF1A = RasGEF domain family member 1A; RELB = RELB proto-oncogene, NF-kB subunit; SHROOM3 = shroom family member 3; SLC17A1 = solute carrier family 17 member 1; SLC26A7 = solute carrier family 26 member 7; SMC2 = structural maintenance of chromosomes 2; STIP1 = stress induced phosphoprotein 1; TMPRSS7 = transmembrane protease, serine 7; TREM2 = triggering receptor expressed on myeloid cells 2; TUBB4A = tubulin beta 4A class Iva; UGDH = UDP-glucose 6-dehydrogenase; ZC3H12A = zinc finger CCCH-type containing 12A.
(TIF)

S1 Table Performance data of animals used for histopathological and gene expression analyses. (XLSX)

S2 Table Characterization of primers used in qRT-PCR. (XLSX)

S3 Table Details of differentially expressed CFP-responsive genes. (XLSX)

S4 Table Details of differentially expressed GLY-responsive genes. (XLSX)

S5 Table Details of enriched signaling CFP-responsive pathways according to KEGG. (XLSX)

## Abbreviations

ACTB: actin beta
ACVP: American College of Veterinary Pathologists
ALDH1_7: alcohol dehydrogenase 1/7
ALDH1A2: aldehyde dehydrogenase 1 family member A2
AMT: aminomethyltransferase
AST: aspartate aminotransferase
ATF5: activating transcription factor 5
A2M: alpha-2-macroglobulin
CBR1: carbonyl reductase 1
CBR3: carbonyl reductase 3
CDH2: cadherin 2
CD59: CD59 molecule
CFH7: complement factor H
CFP: concentrate feed proportion
CON: control
CYP1A1: cytochrome P450, subfamily I (aromatic compound-inducible), polypeptide 1
C5: complement C5
C6: complement C6
DAVID: database for annotation, visualization and integrated discovery
DEG: differentially expressed genes
DM: dry matter
ERRFI: ERBB receptor feedback inhibitor 1
FBN: Leibniz Institute for Farm Animal Biology
FDR: false discovery rate
GC-FID: flame ionization detector gas chromatography
GGT - γ: glutamyltransferase
GLDH: glutamate dehydrogenase
GLY: glyphosate
GO: gene ontology
GPI: glucose-6-phosphate isomerase
GSTA: glutathione S-transferase alpha 3
GSTM3: glutathione S-transferase mu 3
HC: high concentrate
HE: hematoxylin and eosin
KCNC4: potassium voltage-gated channel subfamily C member 4
KEGG: Kyoto Encyclopedia of Genes and Genomes
LC: low concentrate
lfc: log2 fold change
LOD: limit of detection
LOQ: limit of quantification
LURAP1L: leucine rich adaptor protein 1 like
MCFD2: multiple coagulation factor deficiency 2
MF: molecular function
NCBI: National Center for Biotechnology Information
ORAI2: ORAI calcium release-activated calcium modulator 2
PHGDH: phosphoglycerate dehydrogenase
PKIB: cAMP-dependent protein kinase inhibitor beta
PKLR: pyruvate kinase
PLAU: plasminogen activator, urokinase
PLCD4: phospholipase C delta 4
PSEM: pooled standard error of the means
qRT-PCR: quantitative real-time polymerase chain reaction
SLC15A1: solute carrier family 15 member 1
SUCLG2: succinate-CoA ligase GDP-forming beta subunit
SULT1A1: sulfotransferase family, cytosolic, 1A, phenol-preferring, member 1
SULT2A1: sulfotransferase family, cytosolic, 2A, dehydroepiandrosterone (DHEA)-preferring, member 1
t: time
TFRC: transferrin receptor
TG: triglyceride
TKT: transketolase
TMR: total mixed ration
TNFRSF9: tumor necrosis factor receptor superfamily member 9
TPCN2: two pore segment channel 2
TPI: triosephosphate isomerase 1
TUBB4A: tubulin beta 4A class Iva
UXT: ubiquitously expressed prefoldin like chaperone
YWHAZ: tyrosine 3-monooxygenase/tryptophan 5-monooxygenase activation protein zeta
