# Supplementary figures and images for: Effects of glyphosate residues and different concentrate feed proportions in dairy cow rations on hepatic gene expression, liver histology and biochemical blood parameters
Source: PLoS One. 2021 Feb 12;16(2):e0246679. doi: 10.1371/journal.pone.0246679 (PMC7880452; doi:10.1371/journal.pone.0246679)

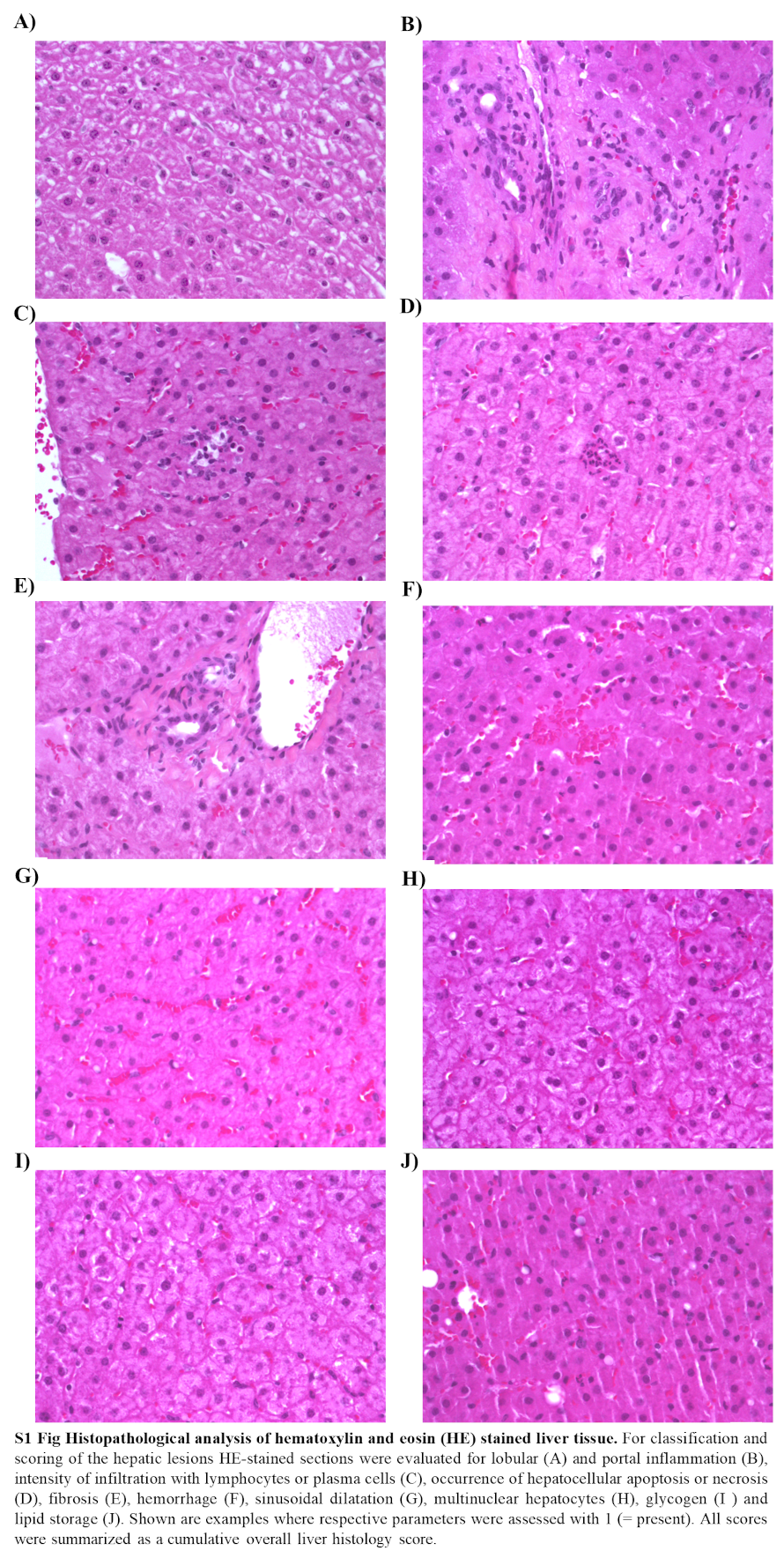

Supplement: S1 Fig — For classification and scoring of the hepatic lesions HE-stained sections were evaluated for lobular (A) and portal inflammation (B), intensity of infiltration with lymphocytes or plasma cells (C), occurrence of hepatocellular apoptosis or necrosis (D), fibrosis (E), hemorrhage (F), sinusoidal dilatation (G), multinuclear hepatocytes (H), glycogen (I) and lipid storage (J). Shown are examples where respective parameters were assessed with 1 (= present). All scores were summarized as a cumulative overall liver histology score. (TIF) [file pone.0246679.s001.tif]

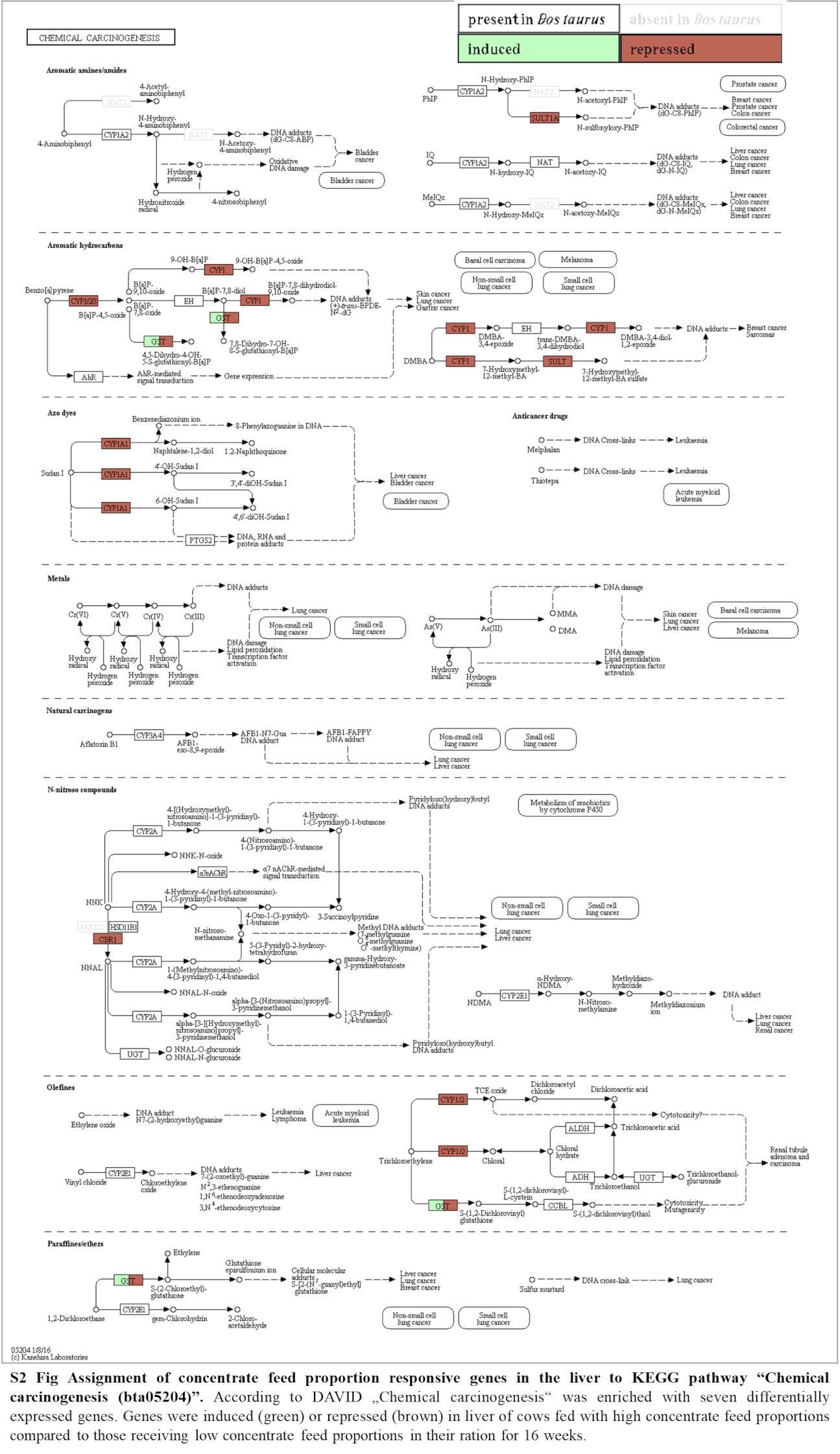

Supplement: S2 Fig — According to DAVID “Chemical carcinogenesis” was enriched with seven differentially expressed genes. Genes were induced (green) or repressed (brown) in liver of cows fed with high concentrate feed proportions compared to those receiving low concentrate feed proportions in their ration for 16 weeks. (TIF) [file pone.0246679.s002.tif]

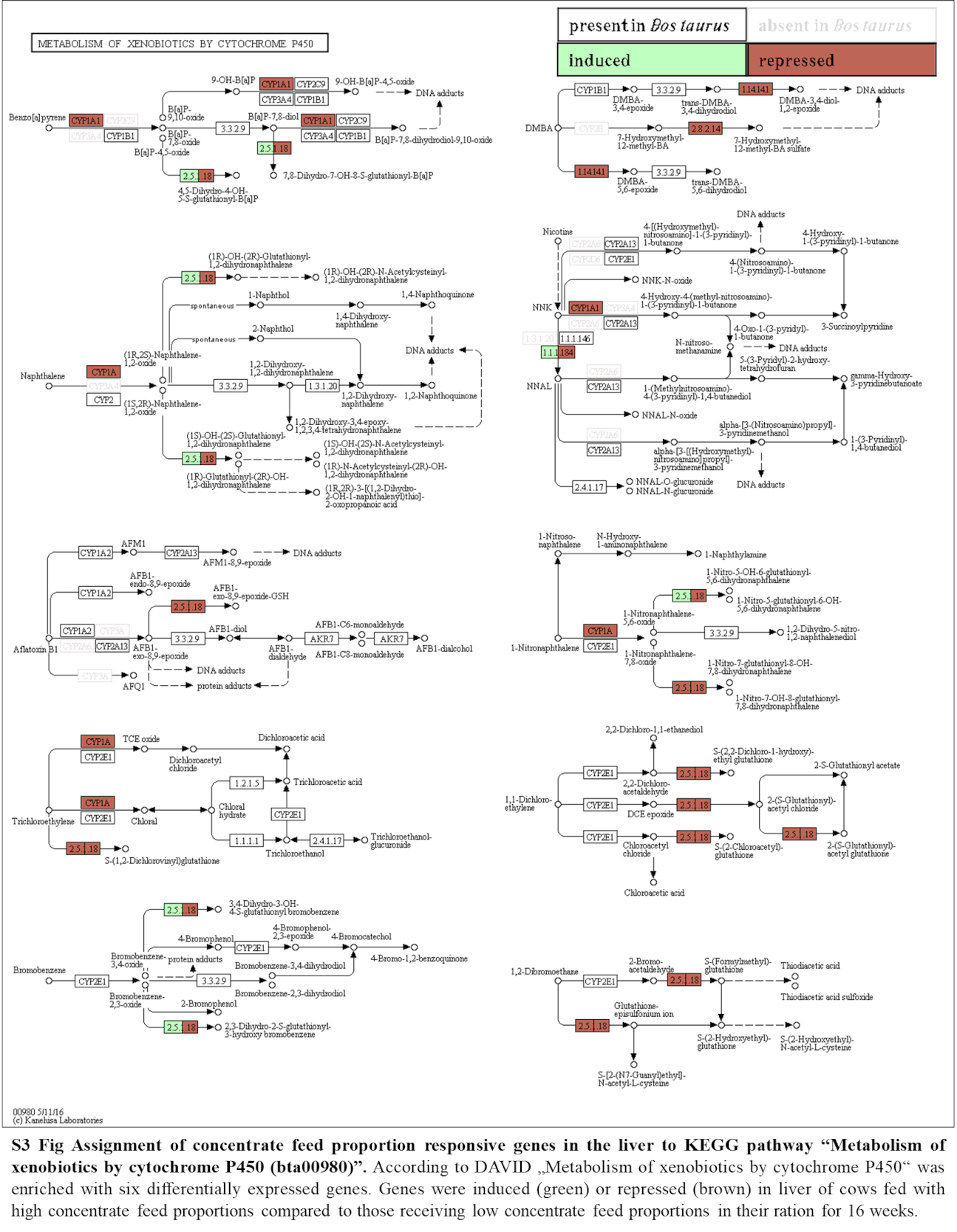

Supplement: S3 Fig — According to DAVID “Metabolism of xenobiotics by cytochrome P450” was enriched with seven differentially expressed genes. Genes were induced (green) or repressed (brown) in liver of cows fed with high concentrate feed proportions compared to those receiving low concentrate feed proportions in their ration for 16 weeks. (TIF) [file pone.0246679.s003.tif]

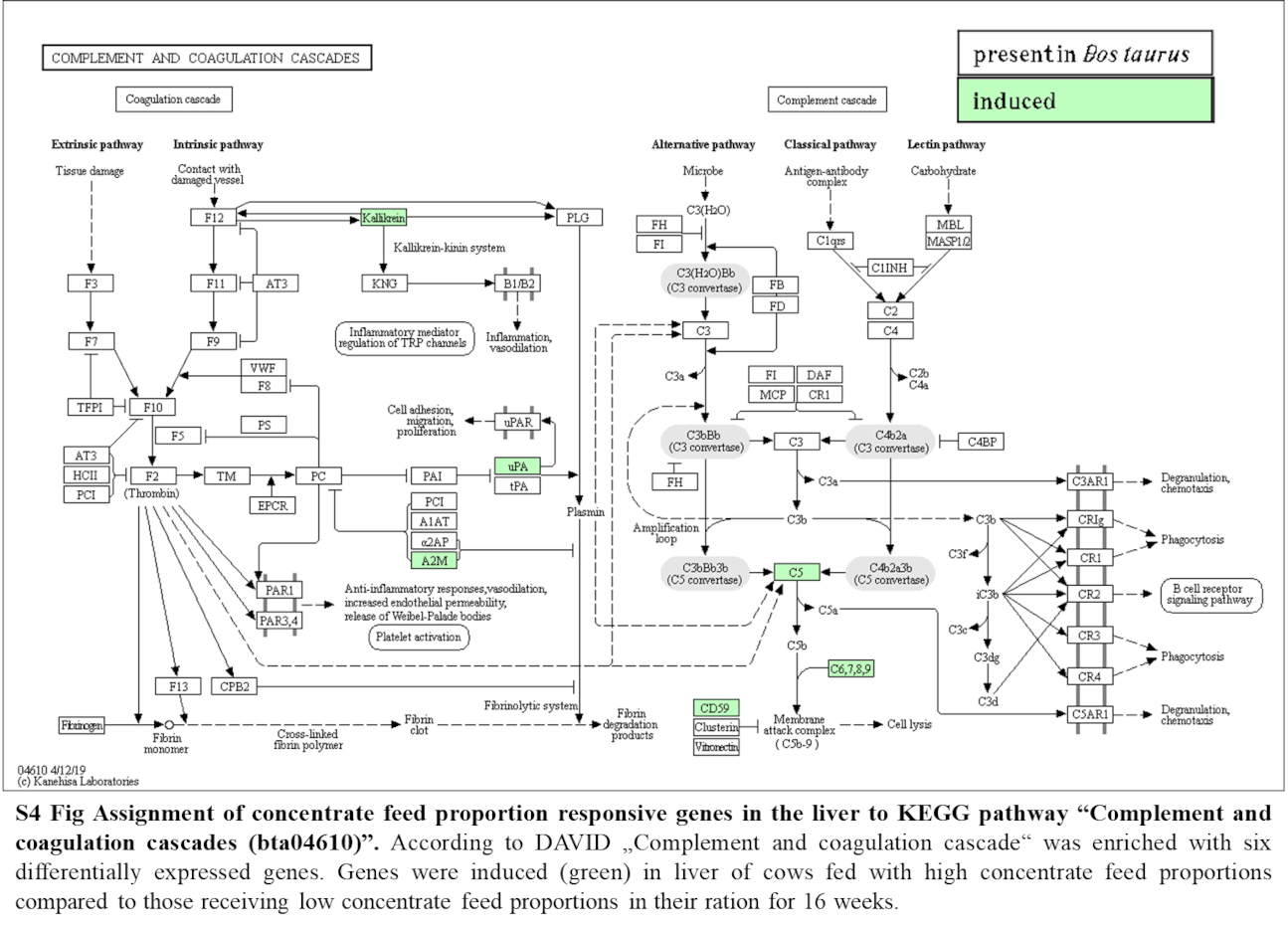

Supplement: S4 Fig — According to DAVID “Complement and coagulation cascades” was enriched with seven differentially expressed genes. Genes were induced (green) or repressed (brown) in liver of cows fed with high concentrate feed proportions compared to those receiving low concentrate feed proportions in their ration for 16 weeks. (TIF) [file pone.0246679.s004.tif]

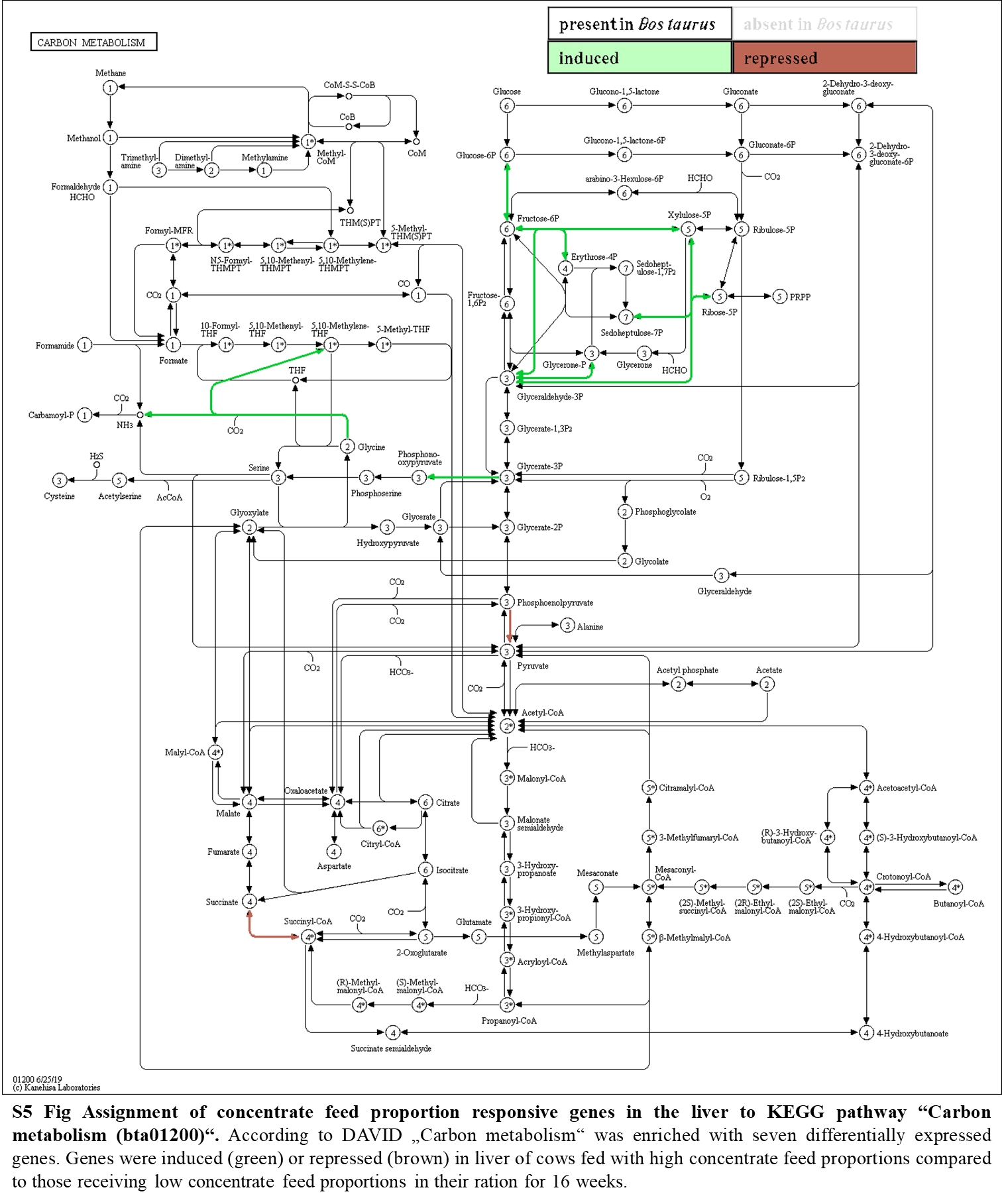

Supplement: S5 Fig — According to DAVID “Carbon metabolism” was enriched with seven differentially expressed genes. Genes were induced (green) or repressed (brown) in liver of cows fed with high concentrate feed proportions compared to those receiving low concentrate feed proportions in their ration for 16 weeks. (TIF) [file pone.0246679.s005.tif]

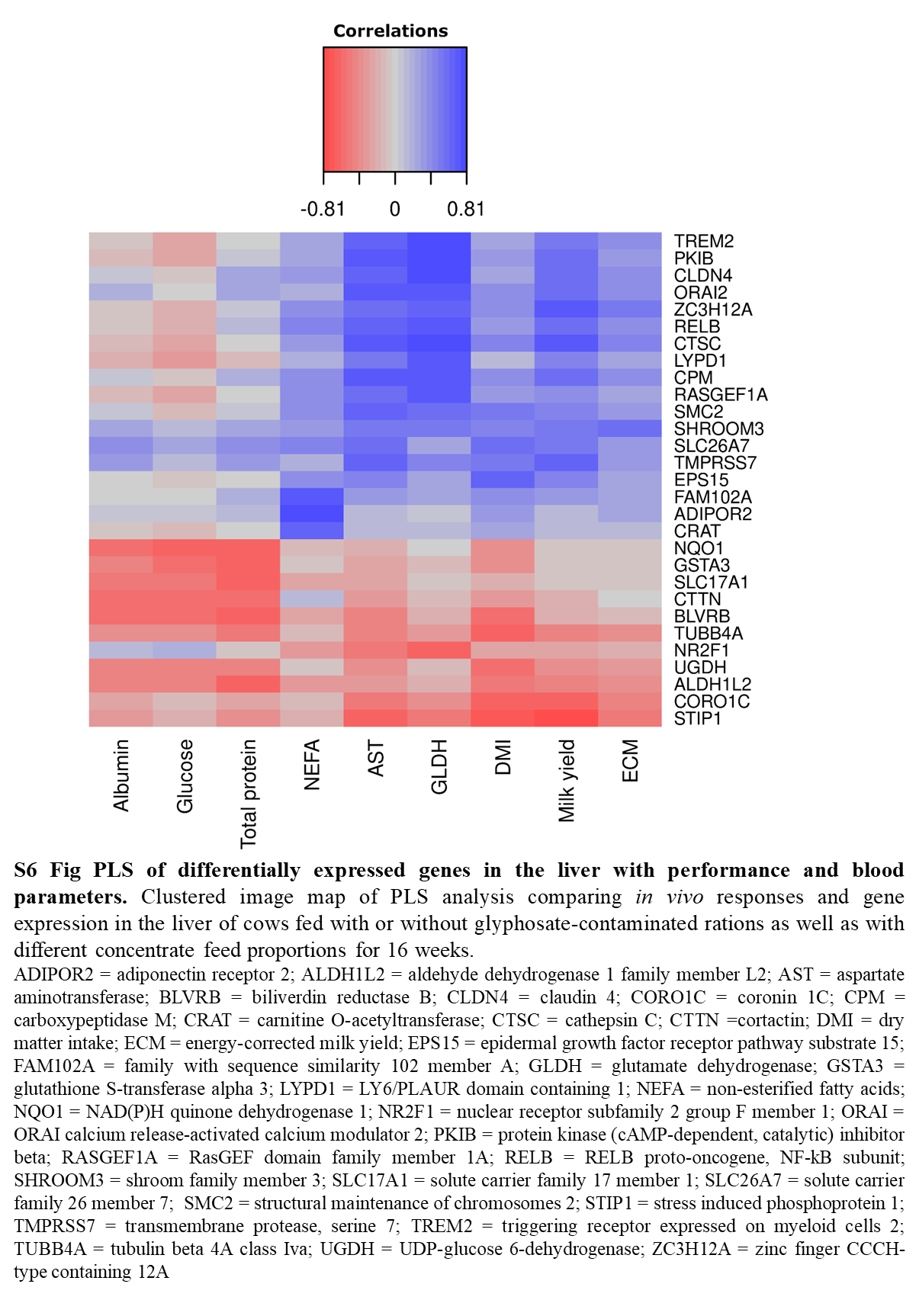

Supplement: S6 Fig — Clustered image map of PLS analysis comparing in vivo responses and gene expression in the liver of cows fed with or without glyphosate-contaminated rations as well as with different concentrate feed proportions for 16 weeks. ADIPOR2 = adiponectin receptor 2; ALDH1L2 = aldehyde dehydrogenase 1 family member L2; AST = aspartate aminotransferase; BLVRB = biliverdin reductase B; CLDN4 = claudin 4; CORO1C = coronin 1C; CPM = carboxypeptidase M; CRAT = carnitine O-acetyltransferase; CTSC = cathepsin C; CTTN = cortactin; DMI = dry matter intake; ECM = energy-corrected milk yield; EPS15 = epidermal growth factor receptor pathway substrate 15; FAM102A = family with sequence similarity 102 member A; GLDH = glutamate dehydrogenase; GSTA3 = glutathione S-transferase alpha 3; LYPD1 = LY6/PLAUR domain containing 1; NEFA = non-esterified fatty acids; NQO1 = NAD(P)H quinone dehydrogenase 1; NR2F1 = nuclear receptor subfamily 2 group F member 1; ORAI = ORAI calcium release-activated calcium modulator 2; PKIB = protein kinase (cAMP-dependent, catalytic) inhibitor beta; RASGEF1A = RasGEF domain family member 1A; RELB = RELB proto-oncogene, NF-kB subunit; SHROOM3 = shroom family member 3; SLC17A1 = solute carrier family 17 member 1; SLC26A7 = solute carrier family 26 member 7; SMC2 = structural maintenance of chromosomes 2; STIP1 = stress induced phosphoprotein 1; TMPRSS7 = transmembrane protease, serine 7; TREM2 = triggering receptor expressed on myeloid cells 2; TUBB4A = tubulin beta 4A class Iva; UGDH = UDP-glucose 6-dehydrogenase; ZC3H12A = zinc finger CCCH-type containing 12A. (TIF) [file pone.0246679.s006.tif]
